# Interplay between the HPA axis and inflammation as mechanisms therapeutic targets of *Cannabis sativa* in depression

**DOI:** 10.3389/fphar.2026.1801474

**Published:** 2026-05-19

**Authors:** Fernanda Pilatti, Marieli Philippsen, Amanda Gollo Bertollo, Cleneide Picinin Webler, Alessandra Kozelinski Bordignon, Zuleide Maria Ignácio

**Affiliations:** 1 Laboratory of Physiology, Pharmacology, and Psychopathology, Graduate Program in Biomedical Sciences, Federal University of Fronteira Sul, Chapecó, Santa Catarina, Brazil; 2 Graduate Program in Neurosciences, Federal University of Santa Catarina, Florianópolis, Santa Catarina, Brazil; 3 Laboratory of Physiology, Research Collaborator, NeuroTCE Group, Pharmacology, and Psychopathology, Federal University of Fronteira Sul (UFFS), Chapecó, Santa Catarina, Brazil

**Keywords:** cannabis, epigenesis, genetic, hypothalamic-pituitary-adrenal axis, major depressive disorder, neuroinflammation

## Abstract

Major Depressive Disorder (MDD) is a highly prevalent and disabling psychiatric disorder, representing a major global health burden across all age groups. Increasing evidence indicates that its pathophysiology involves a complex interplay between chronic stress, dysregulation of the hypothalamic–pituitary–adrenal (HPA) axis, immune activation, and neuroinflammation. Persistent HPA axis hyperactivity, glucocorticoid resistance, and altered expression of key regulators such as FKBP51 contribute to sustained inflammatory signaling and impaired neural plasticity in brain regions involved in mood regulation. Epigenetic mechanisms, including DNA methylation and microRNA-mediated regulation, further modulate stress responsivity, inflammatory pathways, and vulnerability to major depressive disorder. In this context, growing attention has been directed toward *Cannabis sativa* and its bioactive constituents as potential therapeutic agents. Preclinical and clinical evidence suggest that cannabinoids may modulate the endocannabinoid system, attenuate HPA axis hyperactivity, reduce neuroinflammation, and influence monoaminergic and neuroplasticity-related pathways. This review synthesizes the current literature on the mechanistic links among the HPA axis, inflammation, and MDD, highlighting the emerging role of *Cannabis sativa*-derived compounds in targeting these interconnected pathways.

## Graphical Abstract

**Figure:**
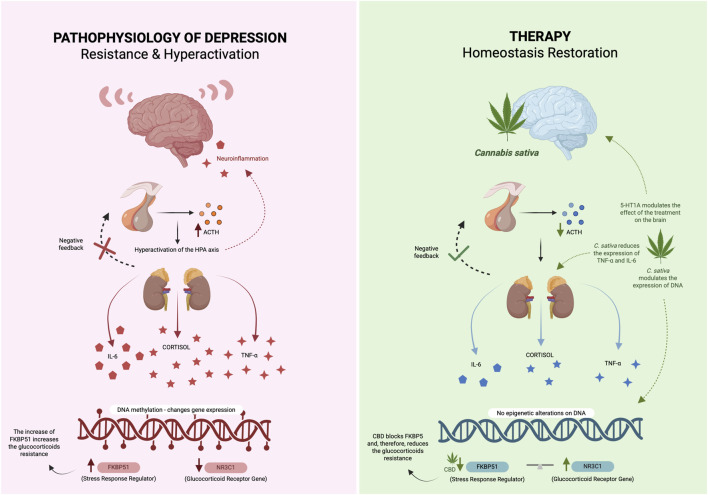

## 1 Introduction

Major Depressive Disorder (MDD) has a high impact on global health. It affects the mental health and well-being of many people, and according to the Pan American Health Organization (PAHO) and the World Health Organization (WHO) (2023), more than 300 million people worldwide of all ages are affected. Recently, the prevalence of depression has increased among young people (Yang et al., 2024; PAHO and WHO, 2026). In the elderly population, the reported incidence of depression was 19.2%, and prevalence may be higher in specific population groups (Jalali et al., 2024). The main symptoms, according to the WHO, are: depressed mood, low self-esteem, fatigue, anxiety, sleep and appetite disorders, loss of interest and pleasure. There is evidence that depression can increase a patient’s risk of developing other health problems (Yang et al., 2024; Kwiatkowska et al., 2025).

The physiological mechanisms involved in the development of depression are complex. Among the various factors involved are alterations related to the hypothalamic-pituitary-adrenal (HPA) axis, inflammation, and neuroinflammation. One of the actions of the HPA axis is to modulate cortisol release, and dysfunction of this system is associated with several pathological conditions, including MDD, post-traumatic stress disorder, anxiety, and metabolic and cardiovascular diseases. According to studies, increased HPA axis activity is observed in situations of chronic stress and plays an important role in neuroinflammation and depression. In MDD, a hyperactive HPA axis is associated with cognitive changes and mood changes (Mikulska et al., 2021; Lei et al., 2025). One of the mechanisms that links chronic stress, inflammation, and depression occurs through the dysregulation of the HPA axis and the activation of immune response patterns. Under normal conditions, cortisol, the primary glucocorticoid secreted by the adrenal glands, reduces inflammation. However, with persistent stress, the HPA axis becomes hyperactivated, leading to excessive glucocorticoid release and dysregulation of its negative feedback loop (Iob et al., 2020; Hassamal, 2023). Glucocorticoids activate receptors throughout the body, and, due to negative feedback failure, they can cause receptor resistance. Studies demonstrate that in patients with MDD, there is hypersecretion of glucocorticoids, resistance to them, and loss of negative feedback, leading to the release of pro-inflammatory cytokines by immune cells (Hassamal, 2023; Kouba et al., 2024).

Neuroinflammation is an abnormal immune response in the brain that involves a cascade of physiological processes triggered by cellular damage. This inflammation and the resulting alterations in brain regions involved in emotional regulation are associated with MDD pathophysiology (Müller, 2014; Herzog et al., 2025). Several cell types mediate neuroinflammation, including microglia, astrocytes, oligodendrocytes, meningeal leukocytes, and peripheral immune cells that selectively penetrate the blood-brain barrier (BBB) and infiltrate inflamed regions (Wu and Zhang, 2023). Several proteins have been studied as markers of neuroinflammatory processes associated with depression, including interleukins, TNF-α, interferons, and translocator protein (TSPO) (Miller, 2020; Troubat et al., 2020; Herzog et al., 2025).

Treating depression, and especially MDD, is a challenge. Psychotherapeutic, psychopharmacological, and interventional psychiatry strategies are used (Havlik et al., 2024). Among the treatments currently being studied is the use of *Cannabis sativa* species, a plant that originated in Central Asia, was domesticated around 12,000 years ago, and has recently been used worldwide, including by the Chinese, Egyptians, Greeks, and Romans (Crocq, 2020). Nowadays, it is a plant widely studied for its chemical diversity and medicinal, industrial, and biotechnological potential. Approximately 566 substances have already been identified, with emphasis on cannabinoids and terpenes, which are responsible for relevant physiological and pharmacological effects, including antioxidant, anti-inflammatory, and antibacterial activities. *Cannabis sativa* shows considerable potential for use, although further studies are needed to understand its properties and safety (Fordjour et al., 2023).

Cannabinoids can be classified into three groups based on their origin: phytocannabinoids, endocannabinoids, and synthetic cannabinoids. Tetrahydrocannabinol (THC) is the primary and most potent psychoactive compound in cannabis. Its medicinal effect is explored due to antiemetic and anti-inflammatory activities, and the ability to reduce neuropathic and chronic pain (Fischedick, 2017). Meanwhile, cannabidiol (CBD) exhibits pharmacological activities, including pain and spasticity control (VanDolah et al., 2019). CBD exhibits a broad spectrum of biological activities, with an emphasis on antioxidant and anti-inflammatory actions, which justifies its study for the prevention and treatment of diseases associated with redox imbalance and inflammation (Sabbag et al., 2025).

Because this review integrates evidence from both human studies and animal experiments, an important distinction must be made between primary MDD in clinical populations and stress-induced depressive-like phenotypes in preclinical models. Animal models are useful for probing mechanisms related to stress responsivity, HPA-axis dysfunction, inflammation, and behavior, but they do not fully recapitulate the diagnostic, clinical, and etiological complexity of primary MDD in humans.

This review presents the main data from the literature on the therapeutic target mechanisms of *Cannabis sativa* in the interaction among the HPA axis, inflammation, and neuroinflammation in the treatment of depression. A translational framework integrating three areas of research often discussed separately is introduced, aiming to conceptually position cannabinoid signaling as a candidate modulator at the intersection of these interacting nodes. Additionally, this review aims to map the current state of the literature and to identify the extent to which the available evidence supports this integrative perspective.

## 2 Hypothalamic-pituitary-adrenal (HPA) axis and depression

Recent literature on depression has shown that observing cortisol alone is not enough to understand how stress affects the organism. Research has highlighted the role of proteins that modulate cortisol sensitivity, particularly FK506-binding protein 51 (FKBP51) and FK506-binding protein 52 (FKBP52), which directly influence the hypothalamic-pituitary-adrenal axis and glucocorticoid receptor function. Studies with animals and humans point to an increase in FKBP51 in chronic stress situations and in people with depression, as reported by Hartmann et al. (2012) and Touma et al. (2011). When this protein is elevated, the receptor responds less efficiently, and negative feedback from the HPA axis is impaired, thereby maintaining the system in prolonged activation.

A clear example of this mechanism appears in the study by Hoeijmakers et al. (2014), in which mice without the FKBP51 gene showed lower corticosterone release, greater receptor sensitivity, and faster recovery after stress. Conversely, animals that possessed the protein exhibited more pronounced HPA axis activation, greater difficulty returning to baseline, and greater behavioral vulnerability to stress. These results reinforce that excess FKBP51 increases the biological risk of depression, while its absence seems to exert a protective effect.

FKBP52 acts in the opposite direction. Riggs et al. (2003) showed that it facilitates nuclear entry of the glucocorticoid receptor (GR), thereby allowing cortisol to function properly. FKBP51, on the other hand, is usually elevated in depressed people and disrupts this process, favoring the emergence of glucocorticoid resistance. In this condition, even with cortisol circulating, the immune system continues to produce inflammatory cytokines, including interleukin-6 (IL-6), interleukin-1β (IL-1β), and tumor necrosis factor-α (TNF-α) as described by Miller et al. (2009) and Slavich and Irwin (2014). These cytokines reach the brain and affect regions implicated in mood, motivation, and energy, contributing to symptoms such as fatigue, persistent sadness, irritability, and reduced motivation.

Thus, the imbalance between FKBP51 and FKBP52 affects not only the hormonal axis but also immune responses to stress, thereby increasing the risk of depressive symptoms. Human studies confirm this link between inflammation, HPA axis dysregulation, and depression. Sharpley et al. (2019) observed that depressive fatigue is more closely associated with systemic inflammation and the cortisol-to-C-reactive protein (CRP) ratio than with isolated cortisol. When the effect of CRP is controlled for, this relationship largely disappears, suggesting that persistent inflammation exerts greater influence than cortisol itself in maintaining symptoms.

Although FKBP51 is essential as a regulator of the glucocorticoid receptor, it becomes problematic when it remains elevated for long periods, whether due to chronic stress, early trauma, or genetic predisposition. In these cases, the organism has difficulty returning to equilibrium, with HPA axis maladjustment and increased inflammation. Overall, this evidence shows that alterations in immunophilins, added to HPA axis dysregulation and persistent inflammation, form a consistent physiological basis for the development and maintenance of depression. This integrative model has been widely accepted because it clearly demonstrates how the stress response, immune function, and glucocorticoid receptor action are deeply connected.

HPA axis regulation exhibits pronounced sexual dimorphism, with distinct endocrine and molecular responses between males and females (Bourke et al., 2012). In experimental models of unpredictable chronic stress, which simulate everyday adversities through random exposure to microstressors, Palumbo et al. (2020) demonstrated that males develop an adaptive mechanism characterized by an increase in cytosolic FKBP51 in the frontal cortex, which contributes to the modulation of glucocorticoid sensitivity. In contrast, females do not exhibit this cytosolic inhibitory response and show impaired regulation of GR nuclear translocation, associated with disorganized localization of FKBP51, which becomes predominantly nuclear (Palumbo et al., 2020). This neurobiological difference is supported by clinical evidence in humans, in which polymorphisms in the FKBP5 gene are associated with increased recurrence of depressive episodes and impairments in HPA axis negative feedback (Moisan, 2021). The reduced expression of GR in limbic regions, associated with FKBP51 dysregulation and hormonal variations of the female reproductive cycle, favors the establishment of a state of resistance to glucocorticoids, resulting in prolonged exposure to glucocorticoids and low-grade inflammation, which supports the higher prevalence and vulnerability of women to the development and maintenance of MDD (Bourke et al., 2012; Palumbo et al., 2020; Moisan, 2021).

## 3 Inflammation and depression

The relationship between inflammation and depression has received increasing attention because many studies show that the immune system plays a central role in how the body and brain respond to stress. Classic research, such as that by Dantzer et al. (2008), has demonstrated that inflammatory cytokines, including IL-6, TNF-α, and IL-1β, can alter brain function and elicit behaviors resembling depression. These molecules, which are supposed to protect the organism, can increase even in the absence of infection, occurring simply under psychological stress. When this happens repeatedly, inflammation ceases to be protective and begins to contribute to symptoms such as extreme fatigue, lack of motivation, difficulty experiencing pleasure, and persistent sadness.

Studies such as those by Miller and Raison (2016) and Slavich and Irwin (2014) showed that individuals with a history of early-life stress exhibit markedly higher levels of inflammation in adulthood. It is a mechanism that helps explain why some people become emotionally exhausted even when their cortisol levels appear normal.

Sharpley et al. (2019) reinforced this same reasoning by showing that depressive symptoms of fatigue were much more linked to inflammation than to isolated cortisol. When the authors controlled for CRP, the association between the cortisol:CRP ratio and fatigue disappeared, indicating that elevated inflammation sustains the symptom. Research by Felger et al. (2016) also shows that inflammation reduces activity in brain regions related to motivation and pleasure. In sum, chronic inflammation alters how the brain responds to stress, modifies emotional circuits, and decreases cortisol sensitivity. It is this set of changes that creates a physiological cycle favoring the development and maintenance of depression.

Codeluppi et al. (2021) investigated the temporal progression of astroglial alterations in the prefrontal cortex under chronic stress. Using transgenic mice and Sholl analysis, the authors demonstrated that prolonged stress exposure (21 and 35 days) induces significant atrophy of astrocytic distal processes. Notably, the study revealed that the reduction in the complexity of these branches directly correlates with the emergence of anhedonia-like behaviors and increased emotionality. These findings, together with the microglial activation and increased TNF-α levels reported by Brás et al. (2022), reinforce the conclusion that neuroimmune dysregulation and the loss of glial structural support are central mechanisms in the pathophysiology of depression.

## 4 Interplay between the HPA axis and inflammation in depression

**FIGURE 1:**
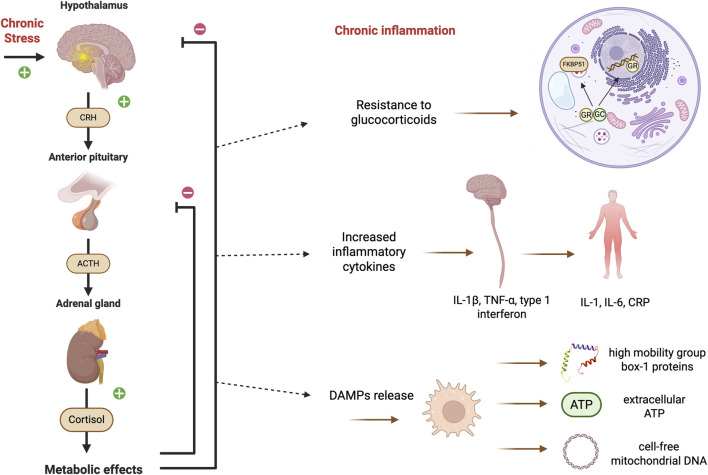
Pathophysiological mechanisms of the HPA axis and chronic inflammation in depression. Chronic stress increases activation of the hypothalamic-pituitary-adrenal (HPA) axis, leading to cortisol hypersecretion. Under physiological conditions, cortisol exerts anti-inflammatory effects through negative feedback on the HPA axis and activation of glucocorticoid receptors (GRs). However, persistent exposure to stress leads to failure of this feedback, GR resistance, and loss of anti-inflammatory control. In parallel, cellular stress induces the release of damage-associated molecular patterns (DAMPs), such as high mobility group box-1 (HMGB1), extracellular adenosine triphosphate (ATP), and cell-free mitochondrial deoxyribonucleic acid (DNA), which increase the production of pro-inflammatory cytokines. Cytokines such as interleukin (IL) IL-1β, tumor necrosis factor-α (TNF-α), and interferons act in the central nervous system, particularly in the hippocampus, reducing neurogenesis and promoting neuronal damage, whereas IL-6, IL-1, and C-reactive protein (CRP) are at higher systemic levels. These cytokines also feed back on the HPA axis, intensifying its activity and reducing glucocorticoid (GC) resistance. A feedback loop is thus formed between hyperactivity of the HPA axis, resistance to glucocorticoids, and chronic inflammation, sustaining neuroinflammation and contributing to the pathophysiology of depression. Additional abbreviations: CRH (corticotropin-releasing hormone); ACTH (adrenocorticotropic hormone); FKBP51 (FK506-binding protein 51). Created with BioRender.

The HPA axis regulates the hormone cortisol, which is responsible for the organism’s response to “fight or flight” stress situations. Dysfunction of this system is associated with several pathological conditions, including depression. Increased HPA axis activity is observed in situations of chronic stress and plays an important role in neuroinflammation and depression. The mechanism linking chronic stress, inflammation, and depression involves dysregulation of the HPA axis and activation of immune response patterns (Figure 1) (Lei et al., 2025; Mikulska et al., 2021).

Under normal conditions, cortisol reduces inflammation; however, with persistent stress, the HPA axis becomes hyperactivated, leading to excessive glucocorticoid release and dysregulation of its negative feedback loop. Due to a failure of this feedback mechanism, resistance to these receptors develops, and in patients with depression, glucocorticoid secretion and pro-inflammatory cytokine release increase (Iob et al., 2020; Hassamal, 2023; Kouba et al., 2024).

MDD patients present higher levels of immunological molecules associated with chronic inflammation. Due to the dysregulation of the HPA axis, several inflammatory cytokines act on the central nervous system (CNS), such as IL-1β, which acts in the hippocampus causing a reduction in neurogenesis, TNF-α, which induces excitotoxic damage in surrounding neurons, and type I interferon, which causes hippocampal damage, resulting in depressive-like behavior (Miller, 2020; Mikulska et al., 2021). Furthermore, studies evidence a relationship between depression and an increase in systemic inflammatory markers such as IL-1, IL-6, and CRP (Lei et al., 2025).

Chronic stress induces cells to release damage-associated molecular patterns (DAMPs), such as high mobility group box-1 proteins (HMGB1), extracellular adenosine triphosphate (ATP), and cell-free mitochondrial deoxyribonucleic acid (DNA), which bind to their own receptors and activate the innate immune response, which in turn activates the inflammasome pathway, where a greater release of pro-inflammatory cytokines occurs (Fries et al., 2023; Hassamal, 2023).

Patients with depression present a higher level of plasma and salivary cortisol than healthy individuals. Untreated or treatment-resistant individuals exhibit increased pro-inflammatory cytokine levels and glucocorticoid resistance (Kouba et al., 2024). Pro-inflammatory cytokines can induce increasing HPA axis activity by modulating GR function and expression (Perrin et al., 2019), thereby contributing to excessive inflammation through GR impairment and chronic cytokine exposure (Pace et al., 2007).

Altogether, the literature indicates that depression is associated with a chronic inflammatory state sustained by the dysregulation of the HPA axis. The interaction among pro-inflammatory cytokines, glucocorticoid resistance, and HPA axis hyperactivity creates a feedback loop that perpetuates inflammation and compromises neural function, particularly in regions associated with mood and neurogenesis. These findings reinforce the central role of inflammation and chronic stress in the pathophysiology of depression and point to the HPA axis as an important therapeutic target (Figure 1).

## 5 Epigenetics in the interplay between the HPA axis and inflammation in depression

**FIGURE 2:**
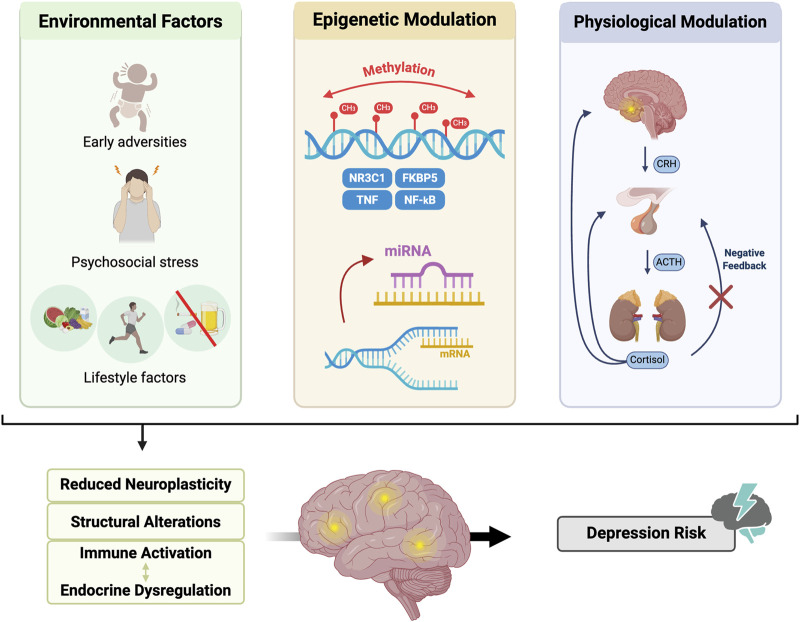
The multi-level interplay in the pathophysiology of depression. The diagram illustrates how environmental factors, such as early-life adversities and psychosocial stress, trigger epigenetic modulation. This includes DNA methylation of key genes (NR3C1, FKBP5, TNF, and NF-κB) and post-transcriptional regulation by miRNAs. These alterations lead to physiological modulation characterized by HPA axis hyperactivation and impaired negative feedback by cortisol. Together, these processes result in immune activation, endocrine dysregulation, and structural brain alterations, ultimately contributing to the development of Major Depressive Disorder (MDD). Created with BioRender.

Because depression is a psychiatric condition characterized by alterations in the HPA axis and inflammatory response regulation, investigating epigenetic mechanisms that modulate these processes is relevant (Figure 2). Methylation of specific genes can influence both the endocrine response and inflammatory pathways. Likewise, microRNAs can mediate the effects of environmental factors on gene expression and contribute to mechanisms of neuroplasticity and immunological regulation.

Epigenetic modifications in key genes related to stress and immunity, such as nuclear receptor subfamily three group C member 1 (NR3C1), TNF, and nuclear factor kappa B (NF-κB), modulate both the negative feedback of the HPA axis and inflammatory pathways mediated by NF-κB, suggesting a link between endocrine dysregulation and immune activation (Palma-Gudiel et al., 2021). In this context, analyses using epigenetic risk scores indicate that DNA methylation patterns associated with chronic inflammation are associated with both the prevalence and incidence of depression (Barbu et al., 2021). Environmental and lifestyle factors strongly influenced these scores, reinforcing the hypothesis that environmental exposures can induce lasting epigenetic alterations as well as contribute to an inflammatory state implicated in the pathophysiology of depression. Although specific miRNAs have been proposed to regulate GR, NF-κB, and cytokine signaling, the current literature does not yet allow a direct link between these interactions to be established, suggesting complex modulatory effects. Thus, mechanistic conclusions regarding the convergence of miRNAs on these pathways remain preliminary. Nevertheless, the observed patterns support the notion that epigenetic regulation can influence stress and immune responses.

Alterations in the GR gene and in the epigenetically sensitive regulators of GR play a significant role. Patients with depression presented increased expression of GR-alpha and reduced expression of FKBP5, accompanied by elevated cortisol levels after the dexamethasone suppression test. The authors argue that this result may be linked to allostatic load, given the long duration of the disorder in the patients evaluated (Hori et al., 2024). Hypermethylation of the NR3C1 exon 1 F region was observed in patients. It was associated with elevated basal and morning cortisol levels, indicating a functional relationship between epigenetic alterations in this gene and HPA axis hyperactivity (Farrell et al., 2018). Complementarily, methylation at specific NR3C1 sites was also associated with cortisol reactivity to stress (Alexander et al., 2018). This profile suggests functional impairment of HPA axis negative feedback, in which GR ceases to perform its physiological regulatory role, possibly due to epigenetic alterations that affect its signaling.

Furthermore, research investigating methylation, gene expression, and neurobiological markers simultaneously found that individuals with MDD showed stronger associations between NR3C1 and FKBP5 cytosine–phosphate–guanine (CpG) methylation and ribonucleic acid (RNA) expression, as well as between hypothalamic structural characteristics and these associations, than healthy controls (Suh et al., 2021). In the same vein, inflammatory epigenetic scores based on CpG methylation, as measured by CRP, were associated with reductions in cortical volume, gray matter, and white matter integrity (Green et al., 2021). The authors suggest that chronic inflammation, epigenetically mediated, may contribute to neurostructural alterations observed in MDD. Concurrently, elevated levels of these inflammatory epigenetic scores at birth and in childhood were identified as predictors of internalizing symptoms throughout development (Barker et al., 2018). This supports the hypothesis that early adversities, inflammation, and psychopathological vulnerability share standard epigenetic bases.

Early-life adversities are frequently implicated as a factor and potential modulator of HPA axis epigenetic modifications. Harsh parenting practices and early childhood stressors were associated with increased methylation of NR3C1 and FKBP5, with measurable effects on the diurnal cortisol rhythm in healthy children (Lewis et al., 2021). Similarly, exposure to childhood maltreatment was associated with hypermethylation of NR3C1 regulatory sites, particularly within the early growth response 1 (EGR1) binding region (Bustamante et al., 2016). Although both depression and abuse are associated with alterations in the methylation of this gene, the location and direction of the effects differ, suggesting partially distinct epigenetic mechanisms.

Based on a longitudinal study, the authors broaden this perspective by showing that methylation of HPA axis genes, including NR3C1, CRH, corticotropin-releasing hormone receptor 1 (CRHR1), and corticotropin-releasing hormone receptor 2 (CRHR2), predicts the risk of MDD during adolescence and early adulthood (Humphreys et al., 2019). The results were independent of genetic variation and family risk and identified sites near functional genomic elements. At the level of specific cytokines, depressed individuals showed lower methylation at a CpG site in the IL-6 gene, whereas antidepressant use was associated with increased methylation at the same site (Ryan et al., 2017). This suggests that methylation of the IL-6 gene may reflect or contribute to inflammatory processes implicated in depression, either as a causal mediator or as a marker of a shared underlying etiology.

Researchers using the dexamethasone suppression test observed that hypermethylation of NR3C1 and, to a lesser extent, of FKBP5, was associated with non-suppression of cortisol and alterations in the corresponding gene expression. However, part of this evidence derives from populations with other psychiatric disorders (Chatzittofis et al., 2021). The authors also emphasize the importance of considering methodological differences, tissue analyzed (blood versus brain), and potential confounding factors, such as early adversities and psychiatric comorbidities.

Post-transcriptional regulation mediated by microRNAs (miRNAs) has also emerged as a component in the epigenetic interface between inflammation, HPA axis functioning, and depression. These small non-coding RNAs modulate complex and dynamic gene networks, allowing environmental signals, such as psychosocial stress, to be translated into changes in gene expression in both the central nervous system and the immune system (Liu et al., 2024).

Patients with MDD disorder exhibited an altered expression profile of circulating miRNAs, with increased levels of hsa-miR-124-3p, hsa-miR-132-3p, hsa-miR-139-5p, hsa-miR-182-5p, hsa-miR-221-3p, hsa-miR-34a-5p, and hsa-miR-93-5p, whereas hsa-miR-144-5p and hsa-miR-135a-5p were reduced compared with healthy controls (Liu et al., 2024). Among these, hsa-miR-124-3p warrants attention for its role in inhibiting synaptic plasticity by negatively regulating the cAMP-response element-binding protein (CREB) pathway and, consequently, serotonin-dependent synaptic facilitation (Liu et al., 2024). These findings suggest that systemic changes in miRNA levels may reflect, at least in part, central neurobiological processes relevant to depression.

miR-124 has also been studied in preclinical models, revealing a context- and brain-region-dependent role. Reducing miR-124 expression in the hippocampus attenuated depressive-like behaviors in rats, which was associated with increased hypothalamic monoamines (norepinephrine, dopamine, and serotonin) and elevated CREB1 and brain-derived neurotrophic factor (BDNF) expression in hippocampal tissue (Yang et al., 2020). Functional analyses in the aforementioned research confirmed that CREB1 and BDNF are direct targets of miR-124, indicating that their silencing can exert antidepressant effects by restoring neuronal plasticity pathways. In adolescent stress models, increased expression of miR-124a and miR-18a was observed in the prefrontal cortex and hippocampus, concomitant with reduced glucocorticoid receptor expression and increased FKBP5 (Xu et al., 2019). These data suggest that miR-124 may also contribute to glucocorticoid resistance and HPA axis dysregulation in specific developmental and stress contexts.

The roles of miRNAs in intercellular communication and HPA axis modulation are diverse, enabling the identification of multiple differentially expressed miRNAs. The injection of small extracellular vesicles derived from patient plasma into the paraventricular nucleus of the hypothalamus (PVN) of mice increased CRF expression. That is, functional evidence that circulating miRNAs can modulate the stress response, as evidenced by genes such as FKBP5 and CRHR2 (Zhu et al., 2025).

In human participants, changes in miRNA expression in peripheral immune cells reinforce the link between inflammation and depression. Intracellular reduction of hsa-let-7e, hsa-miR-146a, and hsa-miR-155 was observed, with levels correlating with the severity of depressive symptoms and partially normalizing after drug treatment (Hung et al., 2019). Given that hsa-miR-146a and hsa-miR-155 regulate inflammatory pathways, these data support an epigenetic mechanism by which chronic inflammation contributes to the pathophysiology of depression. Similar results were observed in another clinical study, in which depressed patients showed increased hsa-miR-342 and reduced hsa-miR-146a and hsa-miR-155 levels in peripheral blood mononuclear cells, with a positive correlation between hsa-miR-342 and TNF-α levels (Brás et al., 2023).

In an experimental study, a chronic social stress animal model induced depressive-like behaviors in mice and significantly reduced miR-184-3p levels in the neurons of the PVN (Xu et al., 2024). The same researchers found that silencing miR-184-3p in the PVN, as well as intranasal administration of its antagonist, mimics the effects of stress, promoting increased CRH biosynthesis and HPA axis hyperactivation. In this model, CRTC1 was identified as a direct target of miR-184-3p, suggesting that the loss of this miRNA compromises molecular mechanisms essential for HPA axis homeostasis and affective behavior regulation (Xu et al., 2024).

The interaction between miRNAs, inflammation, and neuroplasticity also involves microglial mechanisms and inflammasome pathways. The miR-342 has been implicated in microglial activation induced by TNF-α and in NF-kB-dependent inflammatory signaling, processes associated with chronic stress and depressive-like behaviors (Brás et al., 2022; Brás et al., 2020). Complementarily, miR-223-3p exerts neuroprotective effects in the hippocampus, attenuating both neuronal injury and anxiety- and depression-like behaviors induced by chronic unpredictable stress by negatively regulating the NLRP3 inflammasome (Wang et al., 2025). Given that NLRP3 activation promotes the release of pro-inflammatory cytokines, with adverse effects on neural plasticity and neurogenesis, these findings reinforce the role of miRNAs as critical modulators of neuroinflammation associated with depression (Wang et al., 2025).

The diversity of miRNAs involved, their multiple targets, and the dependence on biological and environmental context suggest that we are still facing an expanding field. In other words, miRNAs other than those discussed here likely play complementary or convergent roles in the pathophysiology of depression. Although the field is still expanding, evidence suggests that both endocrine dysfunction and chronic inflammation present modulation mediated by epigenetic alterations, offering potential therapeutic targets for the treatment of depression.

The integration of epigenetic risk scores and DNA methylation patterns suggests the presence of a relatively stable molecular signature associated with chronic inflammation and HPA-axis dysregulation in MDD. As discussed in Section 8, CBD has been reported to influence components of the stress and inflammatory response. However, whether these effects involve epigenetic regulation of genes such as FKBP5 or NR3C1 has not yet been established. Therefore, the possibility that cannabinoid-based interventions could modify the molecular architecture underlying stress responsivity should be considered a hypothesis that warrants rigorous longitudinal and mechanistic investigation in human populations.

## 6 *Cannabis sativa* as a therapeutic strategy for depression

Among preclinical and clinical research, findings point to the therapeutic potential of *Cannabis sativa* and its derivatives in modulating depressive symptoms. However, the literature is heterogeneous with respect to the type of preparation used, cannabinoid profiles, doses, treatment duration, and methodological rigor, all of which influence the interpretation of results. Nonetheless, available studies enable identification of trends in biological mechanisms, symptomatic efficacy, and limitations that inform both clinical use and research agendas.

### 6.1 Evidence in animal models

In this section, the term “animal models” refers mainly to stress-induced or experimentally induced depressive-like phenotypes, rather than to primary MDD itself. These models are valuable for mechanistic investigation, but their translational relevance should be interpreted with caution.

Preclinical studies have shown that the antidepressant effects of *Cannabis sativa* result from interactions among multiple biological systems, including immunological, inflammatory, monoaminergic, and cannabinoid mechanisms (Shin et al., 2024). In animal models, plant extracts have been shown to attenuate depressive-like behaviors and modulate inflammatory markers. Findings indicated a reduction in the neutrophil-to-lymphocyte ratio, regulation of mast cell activation in lymphatic tissues, and inhibition of pro-inflammatory cytokine expression, including TNF-α and IL-1β, in the prefrontal cortex, suggesting that neuroinflammation may represent a relevant pathway underlying the antidepressant-like effects of these preparations (Shin et al., 2024).

Interesting results were observed in a study in which CBD treatment attenuated anhedonia and other depressive-like behaviors, even in the absence of detectable changes in CB1 and 5-HT1 receptor expression, suggesting the involvement of alternative molecular pathways (Lucindo et al., 2025). Subchronic exposure (90 days) to *Cannabis sativa* alkaloid extracts also demonstrated complex and dose-dependent effects. Specific doses attenuated depressive and anxious-like behaviors, while higher doses induced changes in the opposite direction (Fasakin et al., 2022). Biochemical analysis revealed significant alterations in the activities of enzymes involved in neurotransmitter metabolism, including monoamine oxidase (MAO), glutamate dehydrogenase (GDH), angiotensin-converting enzyme (ACE), and acetylcholinesterase (AChE), as well as a dopaminergic response.

Studies with the ethanolic extract of *Cannabis sativa* seeds corroborate the involvement of monoaminergic pathways. Researchers identified increased expression of serotonergic and dopaminergic receptors, as well as elevated brain levels of dopamine, levodopa (L-DOPA), 5-hydroxytryptophan (5-HTP), and serotonin, supporting the hypothesis that modulation of these systems contributes to the observed antidepressant-like effects (Ahn et al., 2021). Consistently, molecular analyses indicate that CBD regulates target genes associated with inflammatory, oxidative, and synaptic processes, suggesting that its therapeutic effects involve anti-inflammatory actions, antioxidant activity, neurotransmitter release, and synaptic plasticity (Shen et al., 2024).

Furthermore, epigenetic and serotonergic mechanisms appear to be involved in these effects. CBD was able to prevent the increase of microRNAs associated with depression in the ventromedial prefrontal cortex in an unpredictable chronic stress induction model, with antidepressant-like effects mediated by the 5HT1A receptor (Bright and Akirav, 2023). In another stress model, it was observed that CBD reversed, in males, alterations in hippocampal CB1 receptor expression, as well as in inflammatory and estrogenic markers. Meanwhile, in females, these effects were not observed, suggesting the recruitment of alternative pathways underlying the compound’s antidepressant-like effects (Bright and Akirav, 2025).

In a study involving exposure to a high-fat diet and chronic stress, the effects of CBD on behavior and on the expression of neuroinflammatory genes in the ventromedial prefrontal cortex and hippocampus were demonstrated (Sabbag et al., 2025). These outcomes varied with the combination of stressors, reinforcing the conclusion that the experimental context decisively influences the observed outcomes.

### 6.2 Evidence in human studies

Clinical and observational studies offer heterogeneous data regarding the antidepressant potential of *Cannabis sativa*. Variability in methods and products used (composition, route, concentration), the lack of standardization, and the tendency of studies to rely on self-reports limit the generalizability and applicability of findings.

Clinical and observational studies evaluating the impact of medicinal cannabis on affective symptoms and sleep presented variable results. A randomized trial comparing immediate versus delayed start of medicinal cannabis use identified significant improvement only in insomnia symptoms, with no detectable effects on anxiety or depression over 12 weeks of follow-up (Gilman et al., 2022). In contrast, evidence from observational studies and case series indicates that the use of cannabis-based medicinal products may be associated with significant reductions in depression and anxiety scores, as well as improvement in sleep quality, observed at different follow-up points, including 1, 3, and 6 months after starting treatment (Mangoo et al., 2022; Wolinsky et al., 2025). Nonetheless, these studies frequently pool heterogeneous cannabinoid profiles, limiting the ability to determine whether observed improvements reflect acute psychoactive effects or sustained antidepressant modulation.

Medium and long-term follow-up data suggest that the clinical response may depend on patients’ initial characteristics. Stability or improvement in anxiety scores was observed over time, with clinically significant reductions among people who received cannabis treatment specifically for anxiety or depression symptoms (Sachedina et al., 2022). In this context, higher symptom levels at the start of follow-up were associated with greater subsequent improvement. However, not all studies report consistent effects; case-control analyses identified inconclusive results: some indicated lower levels of depression, whereas others found no significant differences (Kudahl et al., 2021). The interpretation of these findings was limited by the concomitant use of other psychiatric medications by a substantial portion of the participants.

The use of medicinal cannabis may be associated with a reduction in depressive symptoms, although the effects vary according to the cannabinoid profile and duration of use. Consumption of products with CBD predominance was associated with lower depression scores over time, suggesting a possible role for this compound in symptom modulation (Martin et al., 2021). Similarly, reductions of approximately 50% in perceived symptom intensity were reported with the use of CBD-rich products with low THC content (Cuttler et al., 2018). Despite this, the same study reports a progressive increase in baseline depression levels across sessions, pointing to the possibility of loss of efficacy or even symptomatic worsening with continued use (Cuttler et al., 2018). This finding underscores the need to distinguish short-term symptom relief from durable antidepressant effects, as repeated exposure may not guarantee sustained benefit. In contrast, symptom tracking analyses indicated that acute relief of depressive symptoms was more strongly associated with THC levels, while CBD content did not show a statistically consistent association (Li et al., 2020). These data suggest that THC may contribute more prominently to immediate mood elevation, potentially through its psychoactive properties, whereas its long-term impact on depressive trajectories remains uncertain.

In addition to the effects attributed to isolated cannabinoids, there is a growing body of research on the so-called “entourage effect,” which posits synergistic interactions among the compounds present in the plant. This term describes the interaction between minor cannabinoids and cannabis terpenes, which may enhance the effects of primary cannabinoids such as THC and CBD, resulting in a therapeutic effect superior to that of isolated compounds (Russo, 2019; Ferber et al., 2020; Procaccia et al., 2022; Christensen et al., 2023). Studies have indicated that full-spectrum cannabis products may be more effective than extracts containing isolated cannabinoids across different conditions, including depression (Russo, 2019; Ferber et al., 2020). The combination of multiple compounds may optimize therapeutic outcomes. However, current evidence remains preliminary, and further research is required to elucidate these interactions (Ferber et al., 2020; Christensen et al., 2023). Although some human studies suggest improvements in depressive symptoms with the use of full-spectrum cannabis, the entourage effect hypothesis has not yet been clinically proven and should be regarded as a plausible mechanism rather than a definitive conclusion (Li et al., 2020; Mangoo et al., 2022; Procaccia et al., 2022).

Taken together, the available evidence suggests that the antidepressant potential of cannabis-based products may vary according to cannabinoid profile, dosage, and duration of exposure. While preclinical models provide insights into the molecular pathways through which compounds such as CBD may act, clinical findings remain heterogeneous, and the distinction between transient affective changes and sustained antidepressant responses has not yet been consistently addressed. Some findings also raise the possibility that chronic exposure could be associated with unfavorable mood trajectories. These considerations highlight the importance of cautious interpretation and reinforce the need for further methodologically rigorous investigations, particularly studies that correlate cannabinoid dosage with specific biomarkers of HPA axis activity and inflammatory processes.

## 7 *Cannabis sativa*, inflammation, and depression

In the organism, phytocannabinoids bind to specific receptors distributed throughout various tissues, known as endocannabinoid receptors. These receptors, together with their endogenous neurotransmitters, N-arachidonoylethanolamine (anandamide, AEA), 2-arachidonoylglycerol (2-AG), arachidonoyldopamine (NADA), 2-arachidonoyl glyceryl ether (2-AGE), O-arachidonoilethanolamine, and oleic acid amide (OA), and the enzymes responsible for the synthesis and degradation of endocannabinoids, form the so-called endocannabinoid system (Breijyeh et al., 2021).

The cannabinoid receptors CB1 and CB2 mediate the effects of the endocannabinoids 2-AG and anandamide, acting centrally to modulate synaptic plasticity via retrograde signaling. In this process, 2-AG is synthesized in the postsynaptic neuron and activates presynaptic CB1 receptors, thereby inhibiting neurotransmitter release. Evidence indicates that this mechanism is conserved throughout vertebrate evolution and is involved in learning, locomotion, and dietary control. CB1 is expressed primarily in the CNS, whereas CB2 is found in immune tissues (Elphick, 2012).

CBD exhibits a broad spectrum of biological activities, with an emphasis on antioxidant and anti-inflammatory actions, which justifies its study for the prevention and treatment of diseases associated with redox imbalance and inflammation. CBD can modulate the redox state both directly, by acting on components of the redox system, and indirectly, through interactions with molecular targets associated with this system (Sabbag et al., 2025).

A 2025 study reports that, at the molecular level, CBD treatment modulated nuclear factor kappa B subunit 1 (NFKB1) expression. In female rats exposed to a high-fat diet, CBD reduced the expression of NFKB1 in the ventromedial prefrontal cortex (vmPFC). In the CA1 region, exposure to one or two stressors reduced NFKB1 expression, an effect reversed by CBD treatment, restoring levels to near basal levels (Sabbag et al., 2025).

Previous studies have also demonstrated antidepressant effects of CBD, comparable to those observed with antidepressant drugs. In experimental models, CBD normalized TNF-α overexpression induced by chronic stress in the CA1 region and the ventral subiculum (VS) of male rats. The treatment also restored the reduced expression of CB1, Estrogen Receptors Alpha (ERα) and Estrogen Receptors Beta (ERβ) receptors in the VS, without reversing the changes observed in the vmPFC or the changes in miR-146a-5p expression induced by chronic stress. These findings indicate that the antidepressant effects of CBD are sex-dependent, highlighting the modulation of hippocampal neuroinflammatory and estrogenic pathways in males (Bright and Akirav, 2025).

CBD also induced a potential antidepressant effect in the chronic stress model in male rats. Stress increased depressive-like behavior, evaluated by longer immobility time in the forced swimming test, in addition to altering the expression of microRNAs and genes related to neurotransmission in different brain regions. CBD normalized microRNA expression (miR-16 and miR-135) in the vmPFC and restored induced negative regulation of the HTR1A gene, which encodes the 5-HT1A receptor. Blocking this receptor abolished the antidepressant effect of CBD, indicating that its effects are mediated by the serotonergic pathway and are associated with microRNA modulation in the vmPFC (Bright and Akirav, 2023).

There is growing evidence that CBD is a promising candidate for the treatment of depression, acting through complex mechanisms that involve the modulation of multiple receptors, including CB1, CB2, G-protein-coupled receptor 55 (GPR55), 5-HT1A, and PPARγ (Miao et al., 2025). Administration of CBD in the vmPFC of rats induced antidepressant effects, possibly via indirect activation of 5-HT1A receptors, suggesting that 5-HT1A receptors are involved in the *in vivo* effects of CBD (Russo et al., 2005). Regarding the mechanisms of action of GPR55 and CBD, experimental evidence indicates that CBD attenuates neurobehavioral alterations by inhibiting the lipid lysophosphatidylinositol - G-protein-coupled receptor 55 (LPI–GPR55) signaling pathway; antagonism of this receptor mimics its effects, reducing behaviors associated with depression and anxiety (Zhang et al., 2023).

Acute administration of CBD in the vmPFC of rats reduces immobility time in the forced swimming test, indicating an antidepressant effect. Microinjections in the prelimbic (PL) and infralimbic (IL) subregions were effective, with distinct sensitivities to the drug, and the effects were not due to diffusion to adjacent areas. Blocking CB1 or 5-HT1A receptors prevented the antidepressant action of CBD, indicating that both receptors contribute to this effect in the vmPFC. These findings suggest that CBD modulates serotonergic neurotransmission in the vmPFC by mechanisms dependent on CB1 and 5-HT1A, although evidence of CB1-independent actions also exists (Sartim et al., 2016).

A study conducted by Florensa-Zanuy et al. (2021) showed that CBD reduced cortical activation of NF-κB and IL-6 levels in plasma and the brain, in addition to modulating the KYN/TRP (kynurenine/tryptophan) and KYN/5-HT (kynurenine/serotonin) ratios in the hippocampus and cortex in the lipopolysaccharide (LPS)-induced stress model. These effects were associated with an antidepressant action in the context of neuroinflammation, involving the attenuation of the kynurenine pathway, the inflammatory response, and NF-κB activation (Florensa-Zanuy et al., 2021).

BD antioxidant potential stems from both its molecular structure and its ability to increase the expression of endogenous antioxidant systems, such as superoxide dismutase (SOD) and glutathione peroxidase (GPx), through the nuclear factor erythroid 2-related factor 2 (Nrf2)/Kelch-like ECH-associated protein 1 (Keap1) pathway. Its anti-inflammatory effect is mediated by inhibiting NF-κB activation and pro-inflammatory gene expression, including cytokines and metalloproteinases (Jîtcă et al., 2023).

Therefore, *Cannabis sativa*, primarily through CBD, presents broad therapeutic potential supported by experimental evidence demonstrating antioxidant, anti-inflammatory, anxiolytic, and antidepressant actions. These effects result from the modulation of the endocannabinoid system and multiple molecular pathways involved in neurotransmission, inflammation, and oxidative stress. Despite consistent advances, the findings reinforce the need for additional studies to clarify mechanisms, sex-dependent differences, and safety, consolidating the use of CBD as an evidence-based therapeutic strategy.

## 8 *Cannabis sativa*, HPA axis, inflammation, and depression

Chronic stress is a significant risk factor for various human diseases. Its activation triggers the HPA axis, which is controlled, among other mechanisms, by the GR. GR function is regulated by different proteins, notably the co-chaperone FKBP51 (Scharf et al., 2011).

The FKBP5 gene encodes the FKBP51 protein, which regulates endocrine stress responses by modulating glucocorticoid signaling. Classified as an immunophilin, it has an affinity for immunosuppressants such as FK506 and rapamycin and often exerts antagonistic effects on the actions of its homolog, FKBP52. Its expression is induced by stress and glucocorticoid hormones. Evidence from humans and animal models points to its central role in the physiology of the stress axis. Furthermore, recent studies have expanded its relevance by associating it with metabolic regulation, chronic pain, and the development of stress-related disorders such as depression, PTSD, obesity, and diabetes, consolidating FKBP51 as a potential therapeutic target (Hähle et al., 2019).

Stress responses vary widely among individuals, and understanding their molecular mechanisms is essential for preventive strategies and individualized therapies. In this context, FKBP51 is a central modulator that acts as a co-chaperone, regulating glucocorticoid receptor activity in response to stressors. Additionally, FKBP51 influences various cellular processes in the central nervous system and peripheral tissues (Zannas et al., 2016).

A study characterized basal FKBP5 mRNA expression in the brains of adult male mice. It was induced after treatment with dexamethasone or exposure to various stressors. FKBP5 expression was primarily observed in regions associated with the glucocorticoid receptor, including the hippocampus (with induction in subregions CA1 and the dentate gyrus), the paraventricular nucleus, and the central amygdala. mRNA increases were also observed after restraint stress and food deprivation in the paraventricular nucleus and central amygdala, whereas in the hippocampus, only food deprivation was effective. Regions with low basal expression showed greater induction of FKBP5, reinforcing the role of this gene in modulating glucocorticoid receptor sensitivity (Scharf et al., 2011).

Overexpression of FKBP51 in the basolateral and central amygdala induced stress-like effects and increased anxious behavior in mice. In contrast, selective blocking of FKBP51 by specific antagonists, including SAFit2, reduced the anxiogenic phenotype both after microinjection into the basolateral amygdala and through peripheral administration. These findings, for the first time *in vivo*, demonstrate the central role of FKBP51 and its potential as a therapeutic target for anxiety disorders (Hartmann et al., 2015).

The combination of selective serotonin reuptake inhibitors (SSRIs) and the blocking of FKBP51 showed potential benefits for stress-coping symptoms, while the effects of antidepressants may be impaired by FKBP51 dysfunction. These results suggest the involvement of distinct brain pathways or systems, each modulated differently by the pharmacological association. Furthermore, the suppression of HPA axis reactivity after SAFit2 treatment indicates that behavioral changes induced by SSRIs may be related to modulation of this axis’s function (Pöhlmann et al., 2018).

The previous data corroborate a study, which evaluated corticosterone levels in mice with an FKBP5 gene deletion (FKBP5−/−) and wild-type controls subjected to restraint stress. Basal levels were low in both groups at the start of the diurnal cycle. Following stress, corticosterone increased in all animals, but this increase was attenuated in FKBP5−/− mice. These results indicate that FKBP5 deletion favors glucocorticoid receptor activity, reduces HPA axis reactivity, and contributes to greater resilience to depressive-like behaviors (O’Leary et al., 2011).

In an experimental study, Wang et al. (2023) investigated the interaction between CBD and FKBP5. CBD inhibited the formation of the I kappa B kinase (IKK) complex and the activation of NF-kB, reducing the expression of pro-inflammatory mediators induced by LPS. *In vivo*, oral administration of CBD reduced the overexpression of FKBP51 induced by peripheral nerve injury in activated microglia, suggesting a potential interaction between CBD and FKBP51-related pathways (Wang et al., 2023).

**FIGURE 3:**
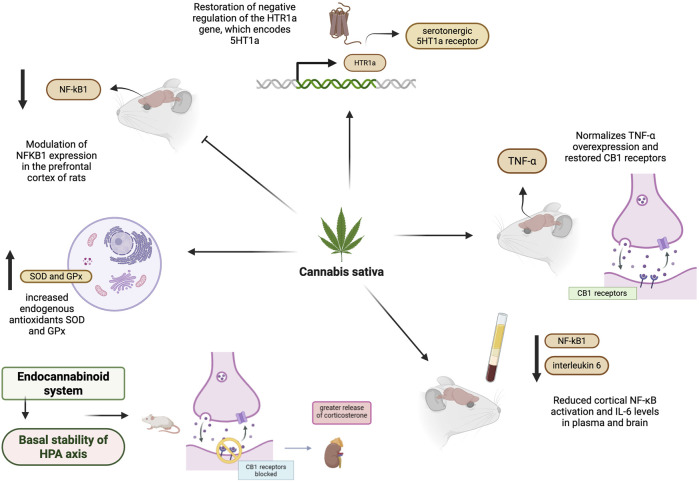
Inhibitory modulation of the HPA axis by the endocannabinoid system. *Cannabis sativa* contains several compounds that interact with receptors of the endocannabinoid system (CB1 and CB2) distributed throughout the central nervous system and tissues. Treatment with CBD modulates central pathways involved in inflammation and neurotransmission. In the inflammatory context, CBD reduced NF-κB pathway activation, as evidenced by decreased NFKB1 expression, reduces cortical NF-κB activation, and normalization of pro-inflammatory cytokines, such as TNF-α and IL-6, across brain regions and in plasma. Simultaneously, CBD restored negative regulation of the HTR1A gene, which encodes the 5-HT1A serotonergic receptor, indicating modulation of the serotonergic pathway implicated in antidepressant effects. The endocannabinoid system helps maintain the basal stability of the hypothalamic-pituitary-adrenal (HPA) axis. Blocking or eliminating cannabinoid type 1 (CB1) receptors intensifies the stress-induced hormonal response, resulting in increased corticosterone release. Created with BioRender.

Altogether, the available evidence suggests that FKBP51 is involved in regulating the HPA axis and stress-related responses, with potential implications for neuroendocrine, behavioral, and inflammatory processes. Altered FKBP51 expression has been associated with anxiety and depression-related phenotypes in experimental and clinical studies. Pharmacological modulation of FKBP51, including through selective antagonists, has been explored in preclinical models, and emerging evidence suggests that CBD may interact with pathways involving this protein, although these mechanisms remain incompletely characterized. The endocannabinoid system also regulates this axis, exercising inhibitory modulation that maintains basal stability (Figure 3).

The endocannabinoid system also regulates the HPA axis. Evidence indicates that it exerts inhibitory modulation on the HPA axis, suggesting that alterations in endocannabinoid tone may be directly related to the development of stress-associated diseases (Cota, 2008).

As demonstrated by Surkin et al. (2018), the endocannabinoid system is a critical neuromodulator involved in pathophysiological processes. The study shows that in male rats previously treated with cannabinoid receptor agonists and antagonists and then subjected to stress, the increase in corticosterone is blocked by anandamide. Additionally, anandamide reduced nitric oxide synthase activity during stress, indicating an anti-stress effect of endocannabinoids at the hypothalamic and adrenal levels. The endocannabinoid system maintains the HPA axis in a stable basal state and attenuates the stress response, thereby allowing recovery of homeostasis (Surkin et al., 2018).

This is corroborated by the study by Patel et al. (2004), in which blocking the CB1 receptor in mice produced a slight increase in basal corticosterone but potentiated the stress-induced increase in corticosterone. In contrast, pharmacological activation by CB1 agonists reduced the hormonal response to stress, indicating that the endocannabinoid system modulates the HPA axis (Patel et al., 2004).

A study showed that mice with the deletion of the CB1 receptor were similar to control animals in low-stress environments but presented increased anxiety and social alterations in unfamiliar contexts. Novelty stress resulted in higher levels of ACTH in animals with the CB1 deletion. This indicates that CB1 signaling modulates behavior in a context-dependent manner and is associated with alterations in HPA axis function (Haller et al., 2004).

The role of the endocannabinoid system is also linked to specific treatments. The deletion of CB1 receptors in mice led to an increase in anxiety-like behaviors and alterations in the stress response, including greater corticosterone release after restraint. Mutant mice presented a reduced response to anxiolytic drugs, such as bromazepam and buspirone, compared to wild-type animals. This indicates that CB1 receptors participate directly in the mechanism of action of anxiolytics, suggesting that dysfunctions in this system may contribute to anxiety disorders and interfere with the efficacy of pharmacological treatment (Urigüen et al., 2004).

In summary, evidence from studies indicates that the endocannabinoid system plays a central role in modulating the HPA axis, functioning as an inhibitory mechanism that maintains basal stability and limits exaggerated stress responses. Alterations in endocannabinoid signaling, particularly involving the CB1 receptor, compromise neuroendocrine homeostasis, intensify stress hormone release, and promote anxious behaviors. Thus, dysfunctions in this system are directly associated with the development of stress- and anxiety-related disorders, in addition to influencing the response to specific treatments (Figure 3).

## 9 Considerations and future directions

This review presents the main aspects of the current literature on interactions and mechanisms linking the HPA axis, inflammation, neuroinflammation, and MDD, with a focus on the role of compounds derived from *Cannabis sativa* in the last decade as therapeutic strategies. The pathophysiology of MDD is complex, with multiple factors contributing to disease development, including the effects of glucocorticoid. One of the control mechanisms for glucocorticoids is the HPA axis, and dysregulation of this axis can occur in chronic stress, neuroinflammation, and depression, where increased HPA axis activity and immune response activation are commonly observed. This results in excessive glucocorticoids release, dysregulation of the negative feedback system, receptor resistance, and the release of pro-inflammatory cytokines.

Cortisol and the proteins that modulate its action, such as FKBP51 and FKBP52, directly influence the HPA axis and GR function. With increased FKBP51expression, as in stressful situations and in MDD itself, the GR responds less efficiently, and negative feedback on the HPA axis is impaired, thereby maintaining the system in prolonged activation. Studies demonstrate that excess FKBP51 increases the biological risk of depression and may favor the emergence of glucocorticoid resistance and the production of inflammatory cytokines. Conversely, the FKBP52 protein acts in the opposite direction, facilitating the nuclear entry of GR, thereby allowing cortisol to function properly. In this way, the imbalance between FKBP51 and FKBP52 affects not only the hormonal axis but also immune responses to stress, thereby increasing the risk of depressive symptoms.

The relationship between depression and inflammation is the target of many studies, which have demonstrated the role played by the immune system in the body’s responses to stress. Inflammatory cytokines, including IL-6, TNF-α, and IL-1β, may have increased expression under psychological stress and can alter brain function, with one consequence being the triggering of depression-like behaviors. Thus, inflammation contributes to symptoms such as extreme fatigue, lack of motivation, difficulty experiencing pleasure, and persistent sadness. In addition to inflammatory cytokines, chronic stress induces cells to release DAMPs, such as HMGB1 proteins, extracellular ATP, and cell-free mitochondrial DNA, which bind to their respective receptors and activate the innate immune response, thereby inducing greater release of pro-inflammatory cytokines.

Depression can also be associated with chronic inflammation, which, in turn, can alter how the brain responds to stress, modify emotional circuits, and decrease sensitivity to cortisol, dysregulating the HPA axis. These findings indicate an important role for inflammation and chronic stress in the pathophysiology of depression and point to the HPA axis as an important therapeutic target. Evidence suggests that both endocrine dysfunction and chronic inflammation can also exhibit modulation mediated by epigenetic alterations, indicating this is an area that may offer potential therapeutic targets for the treatment of depression, requiring further studies.

Evidence from preclinical and clinical studies suggests that *Cannabis sativa*–derived compounds may influence processes associated with depressive symptoms through mechanisms involving the endocannabinoid system and related molecular pathways. Experimental findings, particularly with CBD, indicate modulation of inflammatory mediators, oxidative balance, and neurotransmission, with reductions in depressive-like behaviors reported in animal models. In human studies, however, the evidence remains heterogeneous, reflecting differences in study design, cannabinoid composition, dosage, routes of administration, and treatment duration. Reported effects appear to vary according to cannabinoid profile, including CBD-predominant, low-THC CBD, and full-spectrum products, and interactions among plant-derived compounds remain incompletely characterized. Within this context, the available literature supports a conceptual integration linking cannabinoid signaling with neuroendocrine and inflammatory processes. This perspective aligns with a translational framework integrating research on the HPA axis, inflammation, and neuroinflammation, positioning cannabinoid signaling as a candidate modulator at the intersection of these interacting systems while underscoring the need for further mechanistic and controlled clinical studies.

The findings synthesized in this review indicate that MDD involves a systemic failure of homeostasis within the HPA-Inflammation-Epigenetics triad. One of the important points, the ability of CBD to target and inhibit the FKBP51 protein, is a crucial discovery, as it may facilitate the restoration of GR sensitivity and help re-establish the negative feedback loop of the HPA axis.

Despite the consistent advances over the last decade, these findings and evidence reinforce the need for additional studies to clarify the mechanisms involved, sex-dependent differences, safety, and actions on molecules and binding proteins (e.g., FKBP51). Furthermore, the endocannabinoid system plays an important role in modulating the HPA axis. Evidence indicates that it exerts inhibitory effects on the axis, thereby maintaining basal stability and limiting exaggerated stress responses. Thus, dysfunctions in this system can contribute to the development of stress-related disorders and diseases, as well as influence the response to specific treatments. Further research is needed to elucidate these mechanisms better and, through evidence-based therapeutic strategies, enable the use of *Cannabis sativa*.

Future research should prioritize longitudinal human studies that move beyond self-reports to include objective measurements of FKBP51 expression levels and cytokine profiles (e.g., IL-6, TNF-α, CRP) to quantify “homeostasis restoration.” Furthermore, investigations into the potential reversibility of epigenetic patterns, such as DNA methylation of NR3C1 and FKBP5, through long-term cannabinoid therapy are necessary. Establishing rigorous clinical protocols that consider the interactions among the HPA axis, inflammation, and neuroinflammation, and that rigorously specify the proportions and dosages of cannabinoids, will be essential to consolidate the use of *Cannabis sativa* through a truly evidence-based therapeutic strategy.
